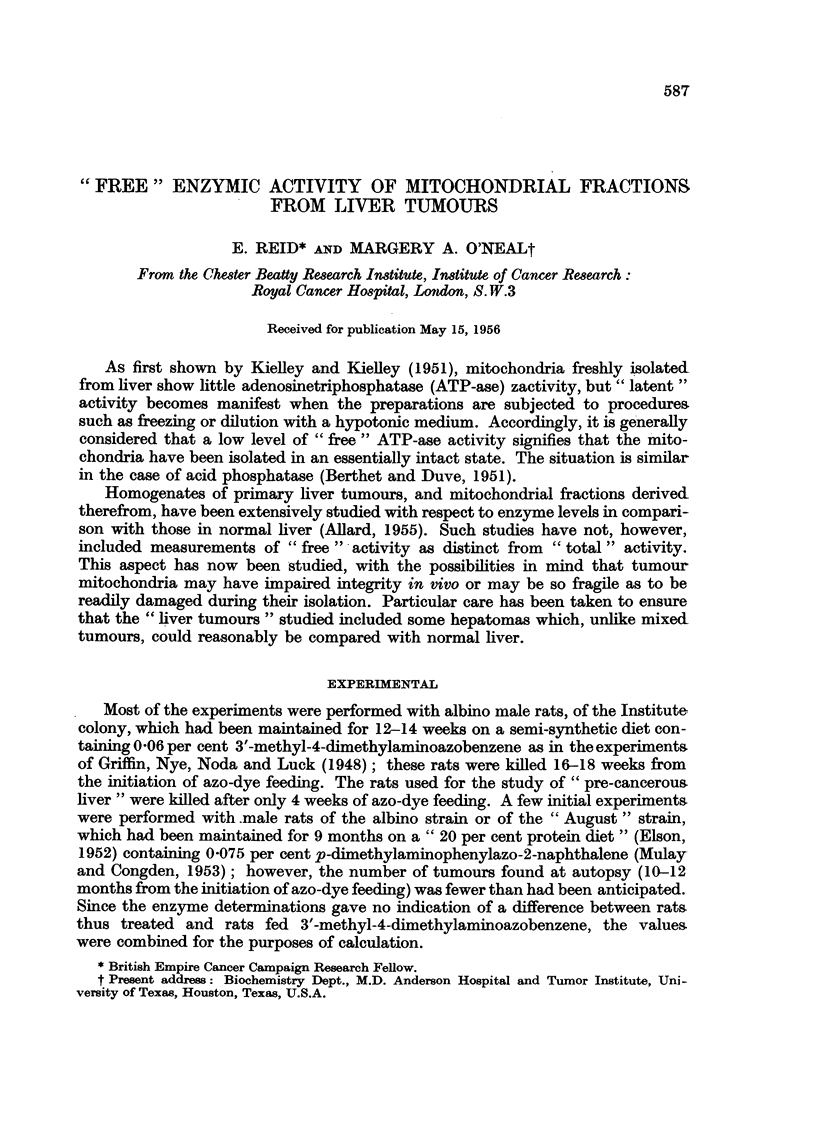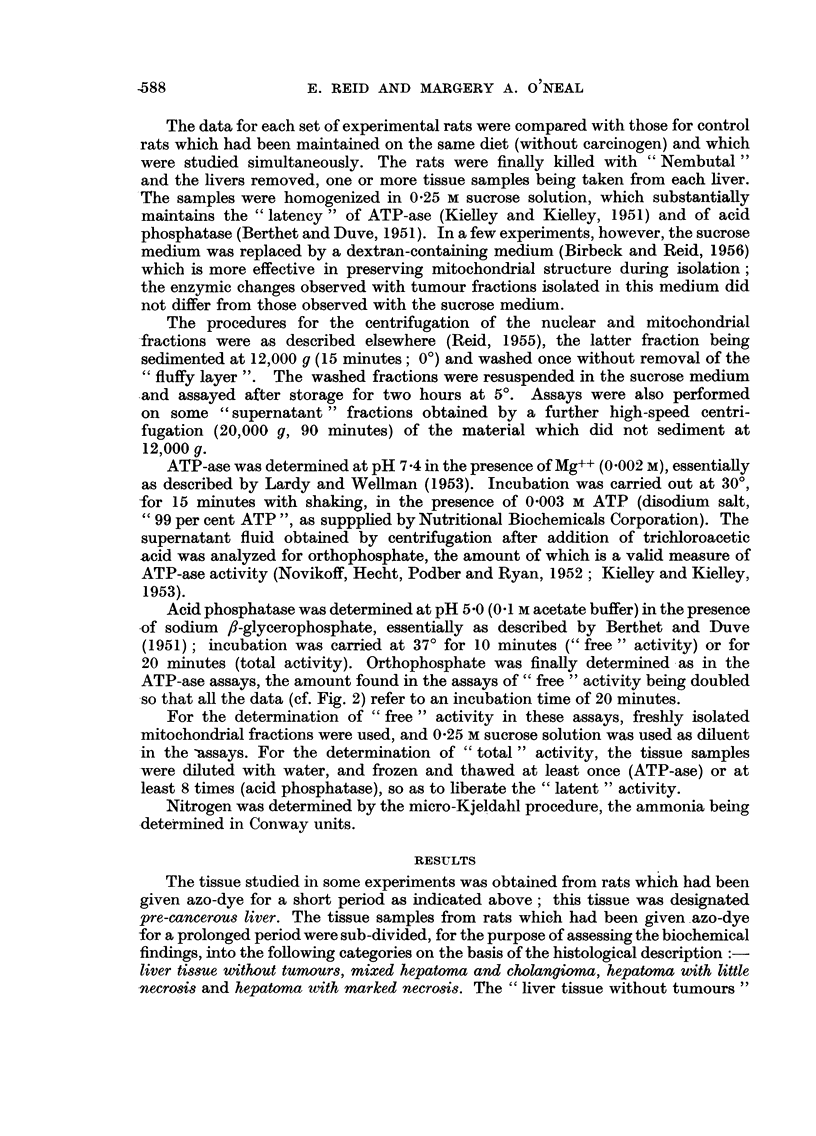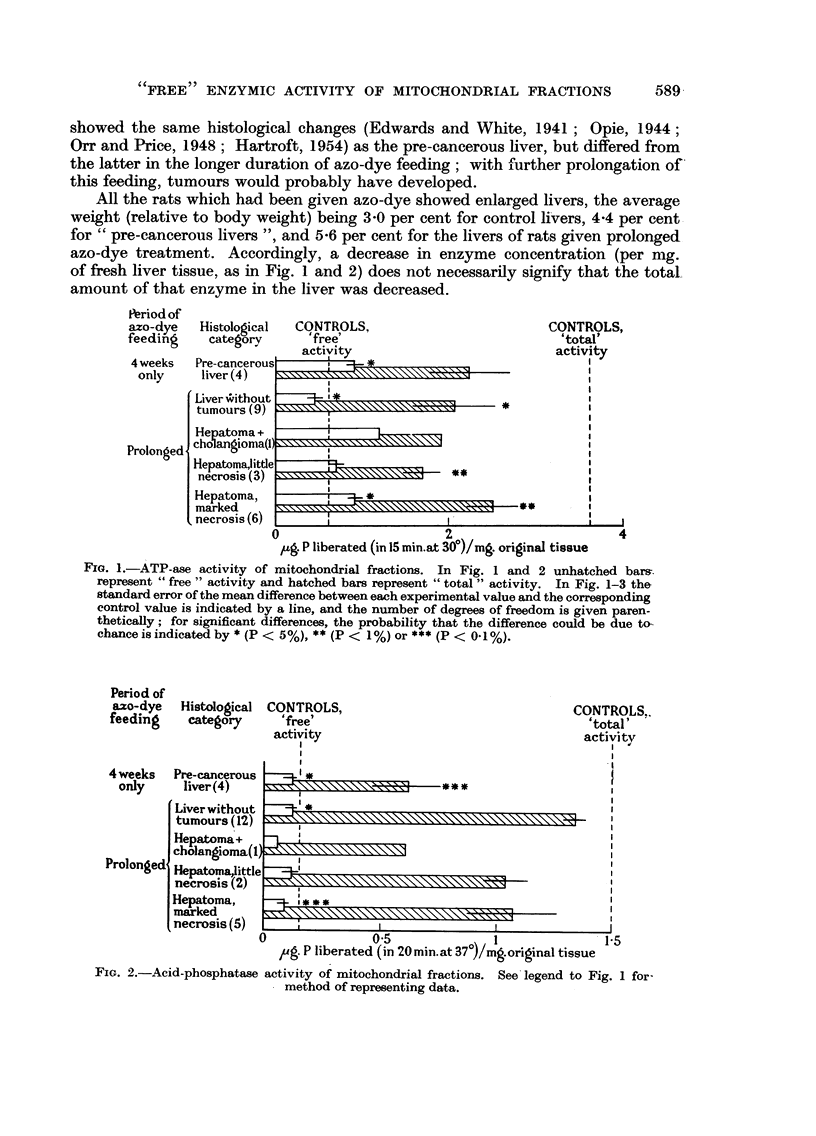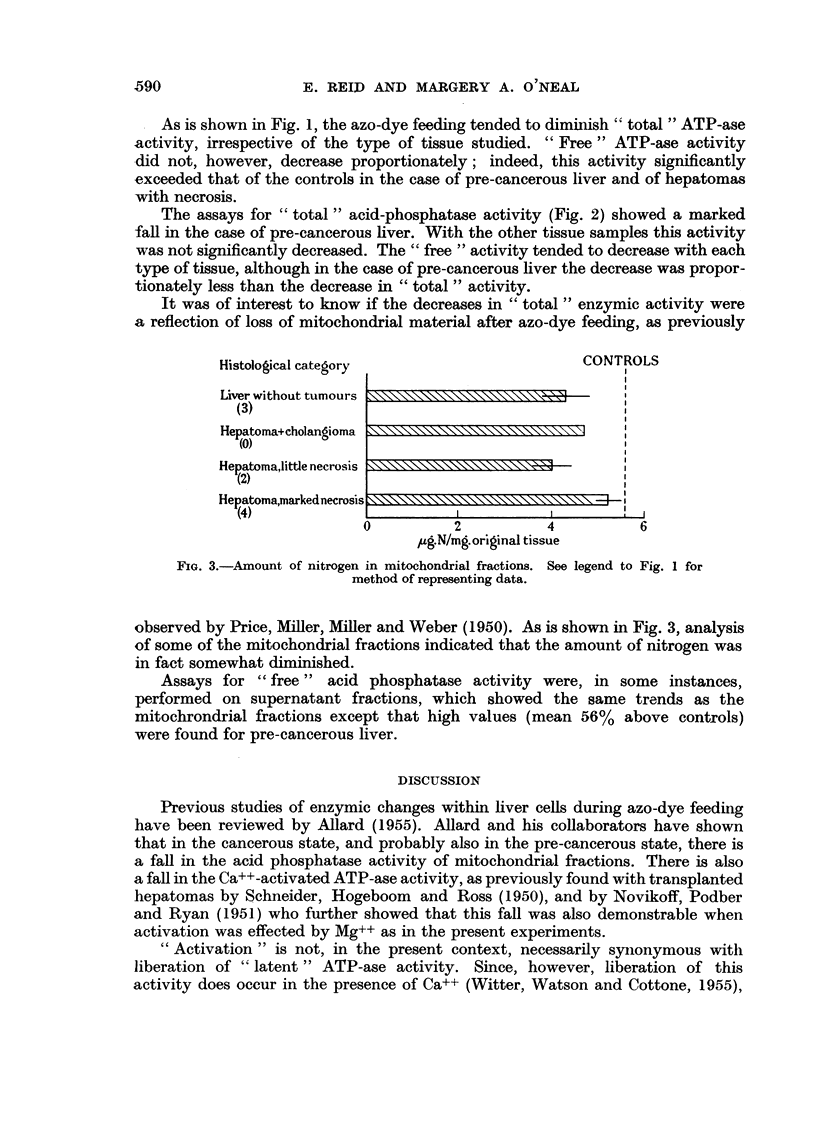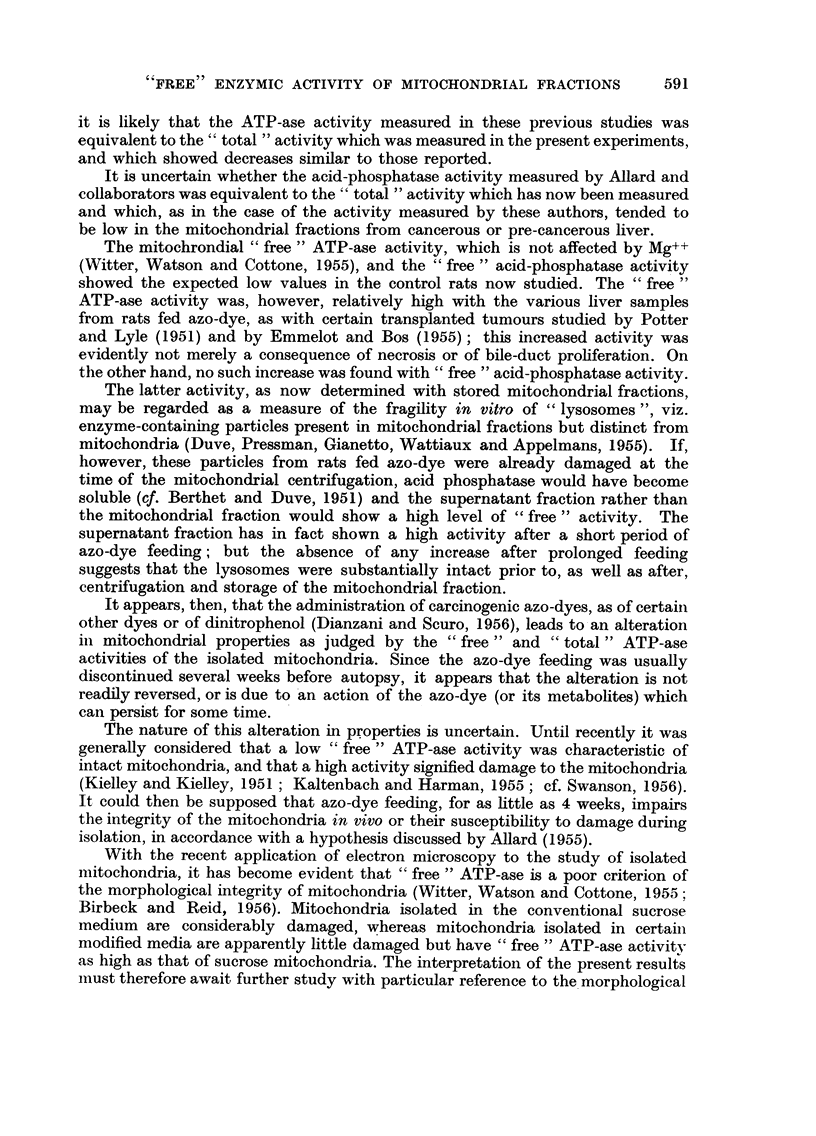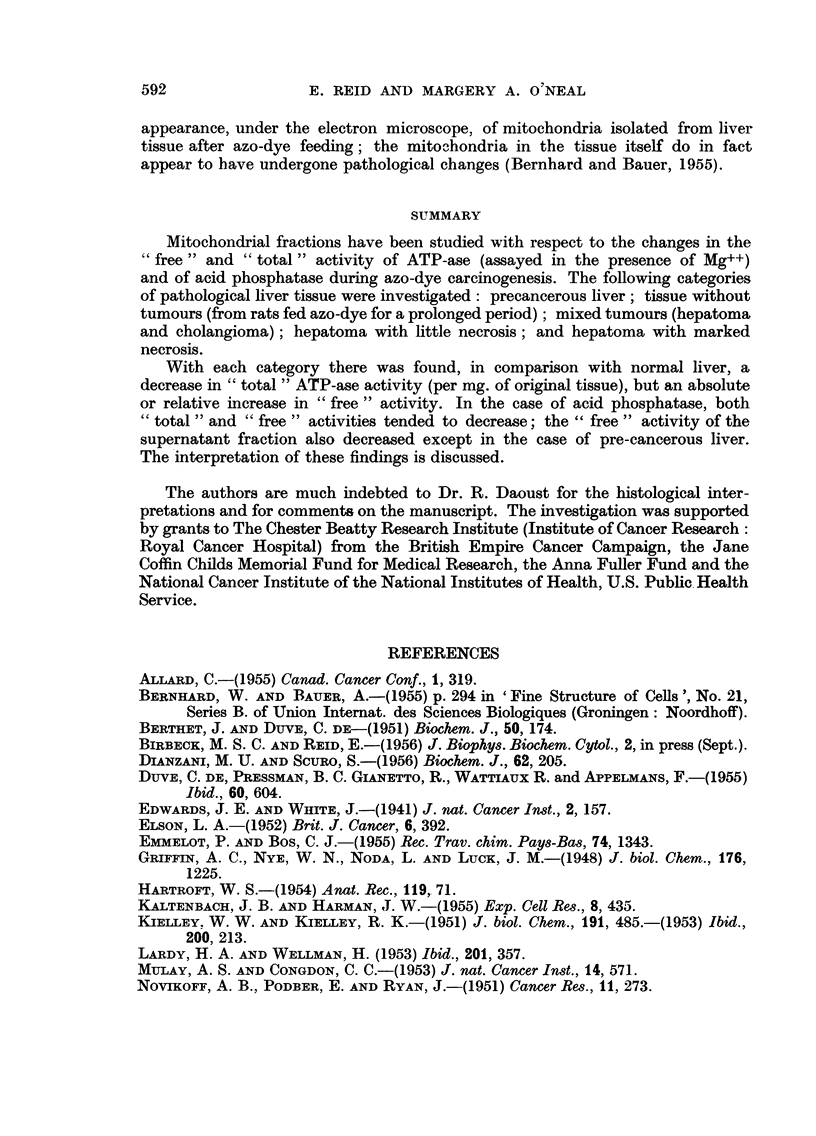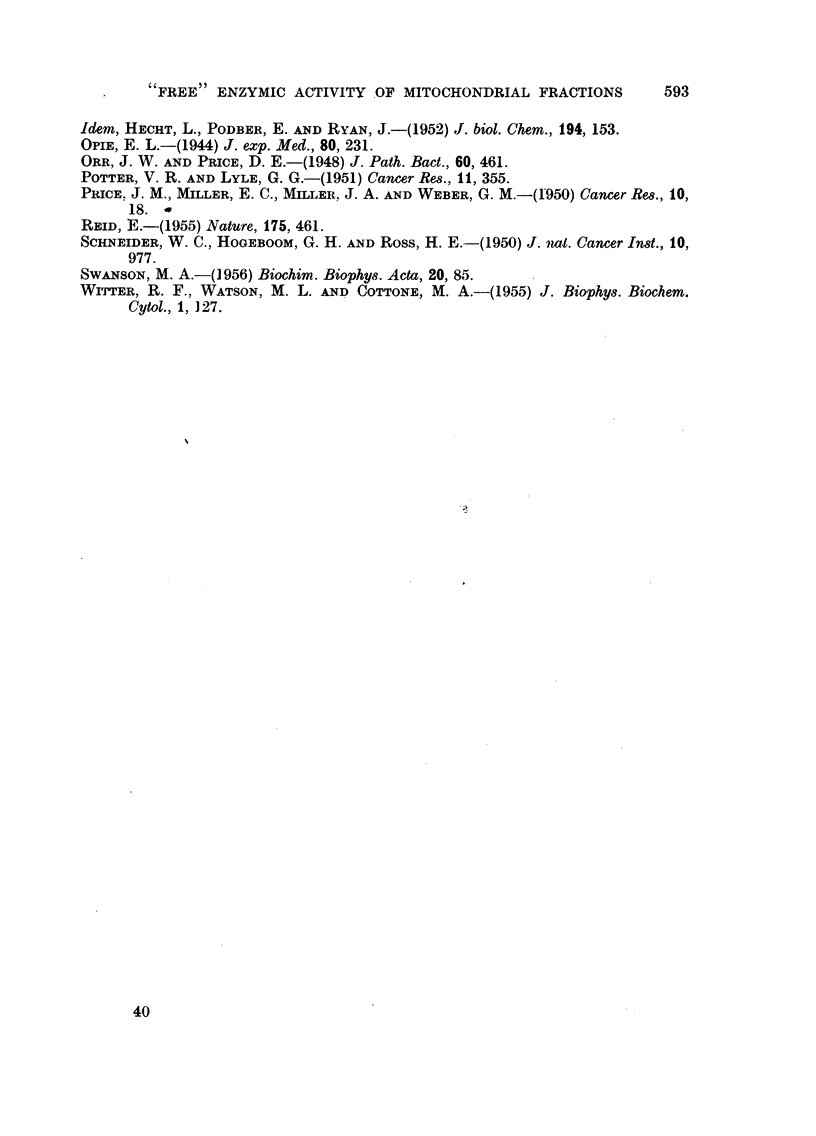# “Free” Enzymic Activity of Mitochondrial Fractions from Liver Tumours

**DOI:** 10.1038/bjc.1956.71

**Published:** 1956-09

**Authors:** E. Reid, Margery A. O'neal


### " FREE " ENZYMIC ACTIVITY OF MITOCHONDRIAL FRACTIONS FROM LIVER TUMOURS

E. REID* AND MARGERY A. O'NEAL†

*From the Chester Beatty Research Institute, Institute of Cancer Research : Royal Cancer Hospital, London, S.W.3*

Received for publication May 15, 1956

As first shown by Kielley and Kielley (1951), mitochondria freshly isolated from liver show little adenosinetriphosphatase (ATP-ase) zactivity, but " latent " activity becomes manifest when the preparations are subjected to procedures such as freezing or dilution with a hypotonic medium. Accordingly, it is generally considered that a low level of " free " ATP-ase activity signifies that the mitochondria have been isolated in an essentially intact state. The situation is similar in the case of acid phosphatase (Berthet and Duve, 1951).

Homogenates of primary liver tumours, and mitochondrial fractions derived therefrom, have been extensively studied with respect to enzyme levels in comparison with those in normal liver (Allard, 1955). Such studies have not, however, included measurements of " free " activity as distinct from " total " activity. This aspect has now been studied, with the possibilities in mind that tumour mitochondria may have impaired integrity *in vivo* or may be so fragile as to be readily damaged during their isolation. Particular care has been taken to ensure that the " liver tumours " studied included some hepatomas which, unlike mixed tumours, could reasonably be compared with normal liver.

### EXPERIMENTAL

Most of the experiments were performed with albino male rats, of the Institute colony, which had been maintained for 12–14 weeks on a semi-synthetic diet containing 0·06 per cent 3′-methyl-4-dimethylaminoazobenzene as in the experiments of Griffin, Nye, Noda and Luck (1948) ; these rats were killed 16–18 weeks from the initiation of azo-dye feeding. The rats used for the study of " pre-cancerous liver " were killed after only 4 weeks of azo-dye feeding. A few initial experiments were performed with male rats of the albino strain or of the " August " strain, which had been maintained for 9 months on a " 20 per cent protein diet " (Elson, 1952) containing 0·075 per cent *p*-dimethylaminophenylazo-2-naphthalene (Mulay and Congden, 1953) ; however, the number of tumours found at autopsy (10–12 months from the initiation of azo-dye feeding) was fewer than had been anticipated. Since the enzyme determinations gave no indication of a difference between rats thus treated and rats fed 3′-methyl-4-dimethylaminoazobenzene, the values were combined for the purposes of calculation.

* British Empire Cancer Campaign Research Fellow.
† Present address : Biochemistry Dept., M.D. Anderson Hospital and Tumor Institute, University of Texas, Houston, Texas, U.S.A.

The data for each set of experimental rats were compared with those for control rats which had been maintained on the same diet (without carcinogen) and which were studied simultaneously. The rats were finally killed with " Nembutal " and the livers removed, one or more tissue samples being taken from each liver. The samples were homogenized in 0·25 M sucrose solution, which substantially maintains the " latency " of ATP-ase (Kielley and Kielley, 1951) and of acid phosphatase (Berthet and Duve, 1951). In a few experiments, however, the sucrose medium was replaced by a dextran-containing medium (Birbeck and Reid, 1956) which is more effective in preserving mitochondrial structure during isolation ; the enzymic changes observed with tumour fractions isolated in this medium did not differ from those observed with the sucrose medium.

The procedures for the centrifugation of the nuclear and mitochondrial fractions were as described elsewhere (Reid, 1955), the latter fraction being sedimented at 12,000 *g* (15 minutes ; 0°) and washed once without removal of the " fluffy layer ". The washed fractions were resuspended in the sucrose medium and assayed after storage for two hours at 5°. Assays were also performed on some "supernatant " fractions obtained by a further high-speed centrifugation (20,000 *g*, 90 minutes) of the material which did not sediment at 12,000 *g*.

ATP-ase was determined at pH 7·4 in the presence of $Mg^{++}$ (0·002 M), essentially as described by Lardy and Wellman (1953). Incubation was carried out at 30°, for 15 minutes with shaking, in the presence of 0·003 M ATP (disodium salt, " 99 per cent ATP ", as suppplied by Nutritional Biochemicals Corporation). The supernatant fluid obtained by centrifugation after addition of trichloroacetic acid was analyzed for orthophosphate, the amount of which is a valid measure of ATP-ase activity (Novikoff, Hecht, Podber and Ryan, 1952 ; Kielley and Kielley, 1953).

Acid phosphatase was determined at pH 5·0 (0·1 M acetate buffer) in the presence of sodium $\beta$-glycerophosphate, essentially as described by Berthet and Duve (1951) ; incubation was carried at 37° for 10 minutes (" free " activity) or for 20 minutes (total activity). Orthophosphate was finally determined as in the ATP-ase assays, the amount found in the assays of " free " activity being doubled so that all the data (cf. Fig. 2) refer to an incubation time of 20 minutes.

For the determination of " free " activity in these assays, freshly isolated mitochondrial fractions were used, and 0·25 M sucrose solution was used as diluent in the assays. For the determination of " total " activity, the tissue samples were diluted with water, and frozen and thawed at least once (ATP-ase) or at least 8 times (acid phosphatase), so as to liberate the " latent " activity.

Nitrogen was determined by the micro-Kjeldahl procedure, the ammonia being determined in Conway units.

### RESULTS

The tissue studied in some experiments was obtained from rats which had been given azo-dye for a short period as indicated above ; this tissue was designated *pre-cancerous liver*. The tissue samples from rats which had been given azo-dye for a prolonged period were sub-divided, for the purpose of assessing the biochemical findings, into the following categories on the basis of the histological description :— *liver tissue without tumours*, *mixed hepatoma and cholangioma*, *hepatoma with little necrosis* and *hepatoma with marked necrosis*. The " liver tissue without tumours "

showed the same histological changes (Edwards and White, 1941 ; Opie, 1944 ; Orr and Price, 1948 ; Hartroft, 1954) as the pre-cancerous liver, but differed from the latter in the longer duration of azo-dye feeding ; with further prolongation of this feeding, tumours would probably have developed.

All the rats which had been given azo-dye showed enlarged livers, the average weight (relative to body weight) being 3·0 per cent for control livers, 4·4 per cent for " pre-cancerous livers ", and 5·6 per cent for the livers of rats given prolonged azo-dye treatment. Accordingly, a decrease in enzyme concentration (per mg. of fresh liver tissue, as in Fig. 1 and 2) does not necessarily signify that the total amount of that enzyme in the liver was decreased.

FIG. 1.—ATP-ase activity of mitochondrial fractions. In Fig. 1 and 2 unhatched bars represent " free " activity and hatched bars represent " total " activity. In Fig. 1–3 the standard error of the mean difference between each experimental value and the corresponding control value is indicated by a line, and the number of degrees of freedom is given parenthetically ; for significant differences, the probability that the difference could be due to chance is indicated by * (P < 5%), ** (P < 1%) or *** (P < 0·1%).

FIG. 2.—Acid-phosphatase activity of mitochondrial fractions. See legend to Fig. 1 for method of representing data.

As is shown in Fig. 1, the azo-dye feeding tended to diminish " total " ATP-ase activity, irrespective of the type of tissue studied. " Free " ATP-ase activity did not, however, decrease proportionately ; indeed, this activity significantly exceeded that of the controls in the case of pre-cancerous liver and of hepatomas with necrosis.

The assays for " total " acid-phosphatase activity (Fig. 2) showed a marked fall in the case of pre-cancerous liver. With the other tissue samples this activity was not significantly decreased. The " free " activity tended to decrease with each type of tissue, although in the case of pre-cancerous liver the decrease was proportionately less than the decrease in " total " activity.

It was of interest to know if the decreases in " total " enzymic activity were a reflection of loss of mitochondrial material after azo-dye feeding, as previously

FIG. 3.—Amount of nitrogen in mitochondrial fractions. See legend to Fig. 1 for method of representing data.

observed by Price, Miller, Miller and Weber (1950). As is shown in Fig. 3, analysis of some of the mitochondrial fractions indicated that the amount of nitrogen was in fact somewhat diminished.

Assays for " free " acid phosphatase activity were, in some instances, performed on supernatant fractions, which showed the same trends as the mitochrondrial fractions except that high values (mean 56% above controls) were found for pre-cancerous liver.

### DISCUSSION

Previous studies of enzymic changes within liver cells during azo-dye feeding have been reviewed by Allard (1955). Allard and his collaborators have shown that in the cancerous state, and probably also in the pre-cancerous state, there is a fall in the acid phosphatase activity of mitochondrial fractions. There is also a fall in the $Ca^{++}$-activated ATP-ase activity, as previously found with transplanted hepatomas by Schneider, Hogeboom and Ross (1950), and by Novikoff, Podber and Ryan (1951) who further showed that this fall was also demonstrable when activation was effected by $Mg^{++}$ as in the present experiments.

" Activation " is not, in the present context, necessarily synonymous with liberation of " latent " ATP-ase activity. Since, however, liberation of this activity does occur in the presence of $Ca^{++}$ (Witter, Watson and Cottone, 1955), it is likely that the ATP-ase activity measured in these previous studies was equivalent to the " total " activity which was measured in the present experiments, and which showed decreases similar to those reported.

It is uncertain whether the acid-phosphatase activity measured by Allard and collaborators was equivalent to the " total " activity which has now been measured and which, as in the case of the activity measured by these authors, tended to be low in the mitochondrial fractions from cancerous or pre-cancerous liver.

The mitochrondial " free " ATP-ase activity, which is not affected by $Mg^{++}$ (Witter, Watson and Cottone, 1955), and the " free " acid-phosphatase activity showed the expected low values in the control rats now studied. The " free " ATP-ase activity was, however, relatively high with the various liver samples from rats fed azo-dye, as with certain transplanted tumours studied by Potter and Lyle (1951) and by Emmelot and Bos (1955); this increased activity was evidently not merely a consequence of necrosis or of bile-duct proliferation. On the other hand, no such increase was found with " free " acid-phosphatase activity.

The latter activity, as now determined with stored mitochondrial fractions, may be regarded as a measure of the fragility *in vitro* of " lysosomes ", viz. enzyme-containing particles present in mitochondrial fractions but distinct from mitochondria (Duve, Pressman, Gianetto, Wattiaux and Appelmans, 1955). If, however, these particles from rats fed azo-dye were already damaged at the time of the mitochondrial centrifugation, acid phosphatase would have become soluble (*cf.* Berthet and Duve, 1951) and the supernatant fraction rather than the mitochondrial fraction would show a high level of " free " activity. The supernatant fraction has in fact shown a high activity after a short period of azo-dye feeding; but the absence of any increase after prolonged feeding suggests that the lysosomes were substantially intact prior to, as well as after, centrifugation and storage of the mitochondrial fraction.

It appears, then, that the administration of carcinogenic azo-dyes, as of certain other dyes or of dinitrophenol (Dianzani and Scuro, 1956), leads to an alteration in mitochondrial properties as judged by the " free " and " total " ATP-ase activities of the isolated mitochondria. Since the azo-dye feeding was usually discontinued several weeks before autopsy, it appears that the alteration is not readily reversed, or is due to an action of the azo-dye (or its metabolites) which can persist for some time.

The nature of this alteration in properties is uncertain. Until recently it was generally considered that a low " free " ATP-ase activity was characteristic of intact mitochondria, and that a high activity signified damage to the mitochondria (Kielley and Kielley, 1951; Kaltenbach and Harman, 1955; cf. Swanson, 1956). It could then be supposed that azo-dye feeding, for as little as 4 weeks, impairs the integrity of the mitochondria *in vivo* or their susceptibility to damage during isolation, in accordance with a hypothesis discussed by Allard (1955).

With the recent application of electron microscopy to the study of isolated mitochondria, it has become evident that " free " ATP-ase is a poor criterion of the morphological integrity of mitochondria (Witter, Watson and Cottone, 1955; Birbeck and Reid, 1956). Mitochondria isolated in the conventional sucrose medium are considerably damaged, whereas mitochondria isolated in certain modified media are apparently little damaged but have " free " ATP-ase activity as high as that of sucrose mitochondria. The interpretation of the present results must therefore await further study with particular reference to the morphological appearance, under the electron microscope, of mitochondria isolated from liver tissue after azo-dye feeding; the mitochondria in the tissue itself do in fact appear to have undergone pathological changes (Bernhard and Bauer, 1955).

### SUMMARY

Mitochondrial fractions have been studied with respect to the changes in the " free " and " total " activity of ATP-ase (assayed in the presence of $Mg^{++}$) and of acid phosphatase during azo-dye carcinogenesis. The following categories of pathological liver tissue were investigated: precancerous liver; tissue without tumours (from rats fed azo-dye for a prolonged period); mixed tumours (hepatoma and cholangioma); hepatoma with little necrosis; and hepatoma with marked necrosis.

With each category there was found, in comparison with normal liver, a decrease in " total " ATP-ase activity (per mg. of original tissue), but an absolute or relative increase in " free " activity. In the case of acid phosphatase, both " total " and " free " activities tended to decrease; the " free " activity of the supernatant fraction also decreased except in the case of pre-cancerous liver. The interpretation of these findings is discussed.

The authors are much indebted to Dr. R. Daoust for the histological interpretations and for comments on the manuscript. The investigation was supported by grants to The Chester Beatty Research Institute (Institute of Cancer Research: Royal Cancer Hospital) from the British Empire Cancer Campaign, the Jane Coffin Childs Memorial Fund for Medical Research, the Anna Fuller Fund and the National Cancer Institute of the National Institutes of Health, U.S. Public Health Service.

### REFERENCES

ALLARD, C.—(1955) *Canad. Cancer Conf.*, **1**, 319.

BERNHARD, W. AND BAUER, A.—(1955) p. 294 in 'Fine Structure of Cells', No. 21, Series B. of Union Internat. des Sciences Biologiques (Groningen: Noordhoff).

BERTHET, J. AND DUVE, C. DE—(1951) *Biochem. J.*, **50**, 174.

BIRBECK, M. S. C. AND REID, E.—(1956) *J. Biophys. Biochem. Cytol.*, **2**, in press (Sept.).

DIANZANI, M. U. AND SCURO, S.—(1956) *Biochem. J.*, **62**, 205.

DUVE, C. DE, PRESSMAN, B. C. GIANETTO, R., WATTIAUX R. and APPELMANS, F.—(1955) *Ibid.*, **60**, 604.

EDWARDS, J. E. AND WHITE, J.—(1941) *J. nat. Cancer Inst.*, **2**, 157.

ELSON, L. A.—(1952) *Brit. J. Cancer*, **6**, 392.

EMMELOT, P. AND BOS, C. J.—(1955) *Rec. Trav. chim. Pays-Bas*, **74**, 1343.

GRIFFIN, A. C., NYE, W. N., NODA, L. AND LUCK, J. M.—(1948) *J. biol. Chem.*, **176**, 1225.

HARTROFT, W. S.—(1954) *Anat. Rec.*, **119**, 71.

KALTENBACH, J. B. AND HARMAN, J. W.—(1955) *Exp. Cell Res.*, **8**, 435.

KIELLEY, W. W. AND KIELLEY, R. K.—(1951) *J. biol. Chem.*, **191**, 485.—(1953) *Ibid.*, **200**, 213.

LARDY, H. A. AND WELLMAN, H. (1953) *Ibid.*, **201**, 357.

MULAY, A. S. AND CONGDON, C. C.—(1953) *J. nat. Cancer Inst.*, **14**, 571.

NOVIKOFF, A. B., PODBER, E. AND RYAN, J.—(1951) *Cancer Res.*, **11**, 273.

*Idem*, HECHT, L., PODBER, E. AND RYAN, J.—(1952) *J. biol. Chem.*, **194**, 153.
OPIE, E. L.—(1944) *J. exp. Med.*, **80**, 231.
ORR, J. W. AND PRICE, D. E.—(1948) *J. Path. Bact.*, **60**, 461.
POTTER, V. R. AND LYLE, G. G.—(1951) *Cancer Res.*, **11**, 355.
PRICE, J. M., MILLER, E. C., MILLER, J. A. AND WEBER, G. M.—(1950) *Cancer Res.*, **10**, 18.
REID, E.—(1955) *Nature*, **175**, 461.
SCHNEIDER, W. C., HOGEBOOM, G. H. AND ROSS, H. E.—(1950) *J. nat. Cancer Inst.*, **10**, 977.
SWANSON, M. A.—(1956) *Biochim. Biophys. Acta*, **20**, 85.
WITTER, R. F., WATSON, M. L. AND COTTONE, M. A.—(1955) *J. Biophys. Biochem. Cytol.*, **1**, 127.